# Nocturnal blood pressure fall as predictor of diabetic nephropathy in hypertensive patients with type 2 diabetes

**DOI:** 10.1186/1475-2840-9-36

**Published:** 2010-08-13

**Authors:** João S Felício, Ana Carolina CB de Souza, Nárcia Kohlmann, Oswaldo Kohlmann, Arthur B Ribeiro, Maria T Zanella

**Affiliations:** 1 Endocrinology Division - UFPA - Universidade Federal do Pará, Belém, Brazil; 2 Endocrinology and Nephrology Divisions - UNIFESP, Universidade Federal de São Paulo, São Paulo, Brazil

## Abstract

**Background:**

Hypertensive patients with reduced blood pressure fall (BPF) at night are at higher risk of cardiovascular events (CVE).

**Methods:**

We evaluated in hypertensive diabetic patients, if a reduced nocturnal BPF can precedes the development of diabetic nephropathy (DN). We followed 70 patients with normal urinary albumin excretion (UAE) for two years. We performed 24-hours ambulatory BP monitoring in baseline and at the end of the study.

**Results:**

Fourteen (20%) patients (GI) developed DN (N = 11) and/or CVE (n = 4). Compared to the remaining 56 patients (GII) in baseline, GI had similar diurnal systolic (SBP) and diastolic BP (DBP), but higher nocturnal SBP (138 ± 15 vs 129 ± 16 mmHg; p < 0.05) and DBP (83 ± 12 vs 75 ± 11 mmHg; p < 0,05). Basal nocturnal SBP correlated with occurrence of DN and CVE (R = 0.26; P < 0.05) and with UAE at the end of the study (r = 0.3; p < 0.05). Basal BPF (%) correlated with final UAE (r = -0.31; p < 0.05). In patients who developed DN, reductions occurred in nocturnal systolic BPF (12 ± 5 vs 3 ± 6%, p < 0,01) and diastolic BPF (15 ± 8 vs 4 ± 10%, p < 0,01) while no changes were observed in diurnal SBP (153 ± 17 vs 156 ± 16 mmHg, NS) and DBP (91 ± 9 vs 90 ± 7 mmHg, NS). Patients with final UAE < 20 μg/min, had no changes in nocturnal and diurnal BP.

**Conclusions:**

Our results suggests that elevations in nocturnal BP precedes DN and increases the risk to develop CVE in hypertensive patients with T2DM.

## Background

Cardiac disease is a common cause of death in patients with DM [1,2]. Predictors of all-causes of mortality in type 1 and 2 diabetics include systolic blood pressure, co-existent cardiovascular disease, and presence of microvascular complications [3-5]. Microalbuminuria is also a marker of cardiac risk and has been related to higher risk of mortality in certain age groups [4].

We have demonstrated that nocturnal systolic blood pressure fall (BPF) is lower in T2DM when compared with essential hypertensives and it is related to left ventricular hypertrophy [6] and diabetic retinopathy [7]. In addition, hypertension and renal impairment in diabetes are linked and it has been reported that hypertensive non-diabetic patients who lack the normal nocturnal decline in blood pressure ('non-dippers') have an increased incidence of cardiovascular complications [8-12]. Therefore, it is a reasonable hypothesis that a reduced BPF during the night could precedes the development of diabetic nephropathy.

Its well known that 24-hours ambulatory BP monitoring (24-h ABPM) has previously identified variations in blood pressure profile in patients with essential hypertension and diabetes and it is related to cardiac events [13-15]. The objective of our study was to evaluate, in hypertensive patients with T2DM, if a reduced sleep BPF predicts the development of diabetic nephropathy.

## Methods

### Patients

The inclusion criteria were: age between 30-70 years, hypertensives with T2DM not treated with insulin before, normal values of urinary protein excretion, creatinine and urinary albumin excretion. The exclusion criteria were: history of congestive heart failure and angina pectoris; cardiac valvulopathy evaluated by echocardiography (ECHO) and history of previous cardiovascular events. Myocardial infarction, stroke, angina pectoris were considered as cardiovascular events. This study was approved by Ethics Committee.

We followed 70 T2DM hypertensive patients (32 males and 38 females) with both normal urinary protein excretion (UPE), urinary albumin excretion (UAE), excretion glomerular filtration rate (eGFR) and serum Cr (Cr), submitted to 24 h-ABPM, during a period of two years. Those patients were recruited from Diabetes and Hipertension ambulatory of Federal University of São Paulo.

At baseline all patients were submitted to 24 h-ABPM, ECHO, UPE, UAE, Cr, fasting blood glucose (FBG), cholesterol (TC), triglycerides (TG) and office BP. Every three months Cr, FBG, TC and fractions, and office BP were repeated. UPE was performed every six months. All patients who developed abnormal proteinuria during the study also had performed UAE. And at the end of the study (2 years follow-up), patients repeated ABPM, UEA and UPE. The development of CVE, and DN, defined as abnormal UPE (confirmed by also abnormal UAE) were considered as end points.

Criteria for established hypertension were systolic and diastolic blood pressure ≥ 140/90 mmHg on repeated measurements [7]. All T2DM patients were treated with diet plus oral hypoglycemic agents. No patient used insulin during the study. Diabetes was diagnosed acoording to the standard criteria. Type 2 DM were identified as those with disease onset at the age of 30 years or after and no need of insulin teatment [6].

The average of FBG, chosen in this study, has been commonly used to monitor glycemic control in type 2 diabetics treated with diet alone or oral hypoglycemic agents. According to several studies, the retrospective average of FBG values is considered a good index to establish a previous long-term glycemic control on those patients [6].

Diabetic Nephropathy was established by microalbuminuria, according to the Gentoften-Montecatini convention [16]. It was considered present when the UAE in a 24-h urine or a short-time collected urine during daytime was in the range of 30-300 mg/24 h (20-200 μ g/min). The upper range was corresponding to a UPE of approximately 0,5 g/l, which was previously considered to be the first marker of clinical diabetic nephropathy [17]. It has been well established that microalbuminuria predicts overt nephropathy in type 1 diabetics. The UAE and UPE are well-documented parts of monitoring those patients [18]. Some surveys also showed benefits on performing these methods in type 2 patients. Microalbuminuria can predicts not only overt renal disease, but also mortality in this population, that is why we opted to perform both methods in this study [17].

#### 24-h Ambulatory blood-pressure monitoring

24-h ABPM was performed using a Spacelabs - 90207 automatic cuff-oscillometric devices (Spacelabs, Inc. Redmond, WA-USA) after 15 days washout of all antihypertensive drugs, after that all subjects restarted on their antihypertensive medications. The monitor was installed in the morning period and it was drop out after 24 hours. The individuals were oriented to keep their regular activities and make a report discriminating the hours of each activity. The device was programmed to perform four measures during each hour. It was established an average systolic and diastolic pressure, during diurnal period, nocturnal period and in 24 hours.

For the purpose of ambulatory blood-pressure monitoring, two different periods were defined. The daytime period included all readings obtained from 8 a.m. until 8 p.m., and the night time period included all readings from 8 pm until 8 a.m. Measures of systolic BP higher than 260 mmHg and diastolic BP higher than 150 mmHg were excluded. The limit to detection of heart rate was between 200 and 20 bpm. The exam was accepted if at least 75% of the measures in 24 hours were successfully executed.

Moreover, it was calculated the nocturnal BPF ({diurnal systolic BP - nocturnal systolic BP} × 100/diurnal systolic BP). It was considered normal values of nocturnal systolic BPF greater than 10% (dippers). Patients that showed BPF lower than this value were called "non-dippers".

#### Echocardiography

M-mode, two-dimensional echocardiographic and cardiac Doppler studies were performed using a commercially available echo-Doppler unit (Esaote Biomedica, Florence, Italy; model SIM 5000) equipped with a 2, 5 MHz mechanical transducer. It was performed with patients in the partial left lateral supine position. M-mode measurements were performed according to the recommendations of American Society of Echocardiography. Left ventricular mass (LVM) was calculated as previously recommended by Devereaux et al [19]. The LVM index was calculated by dividing LVM by the body surface area. All examinations were analyzed by the same echocardiographer that was blinded to the dipping status. Transmitral blood flow signals were obtained on top of mitral valve by apical 4-chamber-view. All measurements of diastolic function were done with normal heart rate (60-100 bpm).

#### Urinary 24 h-proteinuria and serum creatinine

UPE was performed through Kingsbury sulfosalicilic method adapted by Morales and Merino that has normal values below 150 mg in 24 hours. The Cr was determined through Jaffé technique modified by Bartels et al [20].

#### Urinary albumin excretion

UAE was determined by a turbidimetric immunoassay kit. Microalbuminuria was defined by the presence of a UAE rate consistently between 20 and 200 mg/min, as assessed by three 24-h urine samples collected at least 6 weeks after any urinary tract infections or acute hyperglycemic events, and after exclusion of all other causes of albuminuria [21].

#### Long term glycemic control

To create a measure of the previous long-term glycemic control, the average of all FG values available before the study was calculated. If several FG values were recorded during a month, only the first value of the month was used. The same procedure was used to calculate previous indexes of office BP, TC and fractions, and TG. These indices were referred to as the basal average of FG, office BP, TC and fractions, and TG values; and did not differ between the groups.

#### Statistical Analysis

All normally distributed values were given as mean ± SD and all other values were given as median (range). In comparison of the non-normally distributed variables, the Mann-Whitney test was used to test the differences between two groups and Wilcoxon test was used to compare the same groups before and after the follow up period. For all normally distributed variables, the unpaired student's test was used for comparison between two groups and paired student's test was used to compare the same groups before and after. For correlation analysis, correlation coefficients (Pearson or Spearman) were calculated. A P value (two tailed) less than 0.05 was considered statistically significant. All calculations were made with a commercially available program, SigmaStat 1.0 (Jandel Scientific Corporation, Chicago, Illinois).

## Results

Fourteen (20%) patients (GI) developed DN (N = 11) and/or CVE (n = 4). One patient who presented CVE also had DN. Initially, when compared with group of the remaining 56 patients (GII). Group I had similar values of Cr and mean diurnal systolic (SBP) and diastolic blood pressure (DBP) but higher basal levels of both nocturnal SBP (138 ± 15 vs 129 ± 16 mmHg; p < 0.05) and DBP (83 ± 12 vs 75 ± 11 mmHg; p < 0,05) (TABLE 1). In addition, no diference was found between the two groups in relation to the types of antihipertensives used during this study.

**Table 1:** Groups in the baseline according development of cardiovascular events and or diabetic nephropathy during the study

BASELINE	GI (n = 14)	GII (n = 56)	P
Age (years)	57 ± 11	58 ± 8	N.S
BMI (kg/m^2^)	29 ± 5	28 ± 4	N.S
AFBG (mg/dl)	166 ± 38	156 ± 37	N.S
HbA1c (%)	8,6 ± 2,2	8,8 ± 2,5	N.S
DSBP (mmHg)	151 ± 15	144 ± 17	N. S
DDBP (mmHg)	95 ± 12	89 ± 11	N.S
NSBP (mmHg)	138 ± 15	129 ± 16	< 0.05
NDBP (mmHg)	83 ± 12	75 ± 11	< 0.05
SBPF (%)	8 ± 4	10 ± 6	N.S
DBPF (%)	13 ± 6	15 ± 7	N.S

DSBP: Diurnal Systolic Blood Pressure

DDBP: Diurnal Diastolic Blood Pressure

NSBP: Nocturnal Systolic Blood Pressure

NDBP: Nocturnal Diastolic Blood Pressure

SBPF: Systolic Blood Pressure Fall

DBPF: Diastolic Blood Pressure Fall

GI: Patients who presented DN and/or CVE

GII: Patients who did not present DN and/or CVE

AFBG: average of fasting blood glucose values

HbA1c: glycated hemoglobin

Basal nocturnal SBP values correlated with both the occurrence of DN and CVE (r = 0.26; p < 0.05) and with UAE, determined at the end of the study (r = 0,3; p < 0.05).

**Figure 1:**
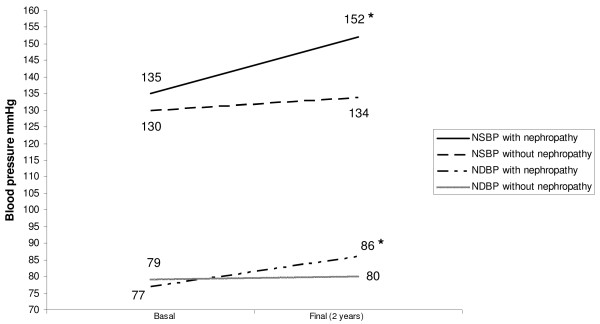
**Variations of nocturnal blood pressure and occurrence of diabetic nephropathy**. * p < 0.05 vs basal. NSBP: Nocturnal Systolic Blood Pressure. NDBP: Nocturnal Diastolic Blood Pressure

**Figure 2:**
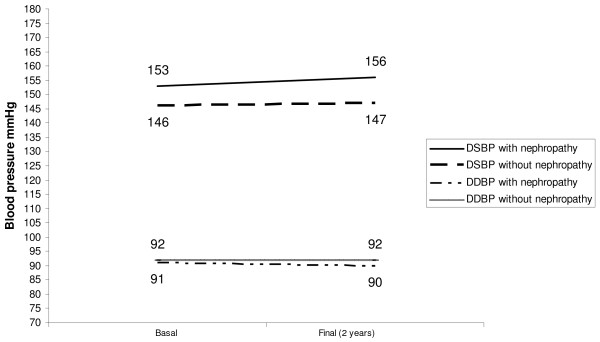
**Variations of diurnal blood pressure and occurrence of diabetic nephropathy**. DSBP: Diurnal Systolic Blood Pressure. DDBP: Diurnal Diastolic Blood Pressure

The basal BPF (%) also showed correlation with final UAE (r = - 0.31; p < 0.05). In patients who developed DN an elevation of nocturnal BP was observed during the study period. In this group, significant reductions were detected in both nocturnal systolic BPF (12 ± 5 vs 3 ±6%, p < 0,01) and diastolic BPF (15 ± 8 vs 4 ± 10%, p < 0,01) while no changes were observed in diurnal SBP (153 ± 17 vs 156 ± 16 mmHg, NS) and DBP (91 ± 9 vs 90 ± 7 mmHg, NS) (TABLE 2, FIGURE 1, FIGURE 2). In the remaining patients, who did not develop DN, with basal and final ABPM determined, no changes were detected in both nocturnal and diurnal BP values and BPF (TABLE 2).

**Table 2:** Variations of blood pressure and occurrence of diabetic nephropathy

	BASAL	GI (n = 11) FINAL	p	BASAL	GII (n = 56) FINAL	P
*DSBP (mmHg)*	153 ± 17	156 ± 16	> 0.1	146 ± 14	147 ± 12	> 0.1
*DDBP (mmHg)*	91 ± 9	90 ± 7	> 0.1	91 ± 12	92 ± 10	> 0.1
*NSBP (mmHg)*	135 ± 12	152 ± 19	< 0.05	130 ± 13	134 ± 19	> 0.1
*NDBP (mmHg)*	77 ± 9	86 ± 10	< 0.05	79 ± 11	80 ± 11	> 0.1
*SBPF (%)*	12 ± 5	3 ± 6	< 0.05	11 ± 4	9 ± 8	> 0.1
*DBPF (%)*	15 ± 8	4 ± 10	< 0.05	11 ± 8	13 + 9	> 0.1

DSBP: Diurnal Systolic Blood Pressure

DDBP: Diurnal Diastolic Blood Pressure

NSBP: Nocturnal Systolic Blood Pressure

NDBP: Nocturnal Diastolic Blood Pressure

SBPF: Systolic Blood Pressure Fall

DBPF: Diastolic Blood Pressure Fall

GI A: Patients who developed DN at the end of the study and also had 24-h ABPM performed after 2 years

GI B: Patients who did not develop DN at the end of the study and also had 24-h ABPM performed after 2 years

## Discussion

In our prospective study, hypertensive normoalbuminuric patients with type 2 DM showed higher basal levels of both nocturnal systolic and diastolic blood pressure preceding the development of CVE and/or abnormal albuminuria. In addition, patients who evolved to micro or macroalbuminuria also showed an increase in nocturnal systolic and diastolic BP and lower BPF at the end of the two years follow up. Patients who remained normoalbuminurics after 2 years did not presented any change in 24-h ABPM profile.

An inversion of day-night BP profile was demonstrated to be related to renal damage in Diabetes [22,23]. Lurbe et al have found that in subjects with type 1 diabetes, elevated systolic BP during sleep precedes the development of microalbuminuria [23]. This abnormality in nocturnal BP should be more a cause than a consequence of diabetic nephropathy. Palmas et al have reported that nocturnal BP elevation predicts progression of albuminuria in elderly people with type 2 Diabetes [24]. As we are aware, our study is the first to demonstrate that an elevation of both nocturnal systolic and diastolic BP could also precede microalbuminuria in hypertensives withT2DM.

Autonomic neuropathy (AN) could be a via through hyperglycemia would elevate nocturnal SBP levels. Some studies have related AN to an increase of left ventricular mass and to the loss of nocturnal BP decrease [25]. Other conditions, such obstructive sleep apnea and renal failure with fluid retention might be involved [26]. Whatever its mechanisms might be, the increase of systolic nocturnal BP and or lack of normal nocturnal BP fall have been related to chronic complications in diabetic patients [27].

There are a few limitations of 24-h ABPM technique in diabetic patients. One of the main problems is the necessity of validation studies of each oscillometric device. We recently tested and retested the Spacelabs - 90207 automatic cuff-oscillometric devices (Spacelabs, Inc. Redmond, WA-USA) in hypertensive type 2 diabetic patients [28] and we found that it is a reliable method to clinical management of those patients. An obstacle that remains is the high price and limited availability of 24-h ABPM devices in general practice. Another problem is the definition of 24-h ABPM cut-offs for hypertension diagnosis and therapeutic goals in type 2 DM. We recently also described that diabetic patients with nocturnal systolic BP ≥ 140 mmHg had a great increase on prevalence of left ventricular hypertrophy (LVH) [6]. Furthermore, Staessen et al [29], in a meta-analysis of 23 studies, including a total of 3,476 normal subjects, concluded that only levels ≥ 137 mmHg for nocturnal SBP should be considered as definite hypertension. This nocturnal SBP value is very close to the level that we found to start increasing the risk of LVH. Unfortunately, our relative small number of patients did not allowed to calculate thresholds and achieve a consistent multivariate analysis

According to Parati et al [30], a noninvasive and powerful test such as 24-h ABPM could be useful in every diabetic subject. However, given the limited availability of this test in daily practice, the author suggests that performing 24-h ABPM or not, should be evaluated on case-by-case basis, evaluating some key and risk factors such previous clinical events (stroke and myocardial infarction) and the presence of impaired renal function. In our opinion, the presence of hypertension *per se *in diabetic patients must be an indication to perform 24 h-ABPM. The cost must be reduced to that approach be possible.

It has been pointed out the importance of cardiometabolic risk factors in hypertensive patients [31]. Belletti et al found that as compared to hypertensive patients without any additional cardiometabolic risk factors, those with diabetes, obesity and hyperlipidemia were less likely to have controlled BP. In our study, the groups did not differ in body mass index, diabetes control and in the kind of antihypertensive treatment. In addition, the relation between hyperlipidemia and development of diabetic nephropathy is not clear. Nevertheless, it is possible that some of these variables, as confounders, could be involved in elevation of nocturnal blood pressure and evolution to diabetic nephropathy.

## Conclusions

In summary, our study suggests that an elevation of nocturnal systolic blood pressure and a loss of nocturnal blood pressure fall might precede the development of diabetic nephropathy and cardiovascular events in hypertensive normoalbuminuric patients with type 2 diabetes. Other studies are necessary to confirm these findings.

## Competing interests

The authors declare that they have no competing interests.

## Authors' contributions

JF conceived the study, participated in its design and coordination and performed the statistical analysis. AS, NK and OK participated in the sequence algnment. AR and MZ participated in the designed of the study. All authors read and approved the final manuscript.

## References

[B1] Sturrock NDC George E Pound N Stevenson J Pech GM Sowter H Non-dipping circadian blood pressure and renal impairment are associated with increased mortality in diabetes mellitus Diabetic Medicine 2000 17 360 364 10.1046/j.1464-5491.2000.00284.x 10872534

[B2] Waugh NR Dallas JH Jung RT Newton RW Mortality in a cohort of diabetic patients Diabetologia 1989 32 103 104 10.1007/BF00505181 2721839

[B3] Farmer CKT Goldsmit DJA Quin JD Dallyn P Cox J Kingswood JC Sharpstone P Progression of diabetic nephropathy--is diurnal blood pressure rhythm as important as absolute blood pressure level? Nephrology Dialisis Transplant 1998 13 635 639 10.1093/ndt/13.3.635 9550639

[B4] Verdecchia P Schillaci G Gatteshi C Zampi I Battisnelli M Bartoccini C Blunted Nocturnal Fall in blood pressure in hipertensive women with future cardiovascular morbid events Circulation 1993 88 986 922 8353926 10.1161/01.cir.88.3.986

[B5] Borch-Johnson K Andersen PK Deckert T The effect of proteinuria on the relative mortality in type 1 (insulin-dependent) diabetes mellitus Diabetologia 1985 28 590 596 4054448 10.1007/BF00281993

[B6] Felício JS Pacheco JT Ferreira SR Plavnik F Moisés VA Kohlmann O Ribeiro AB Zanella MT Hyperglycemia and nocturnal systolic blood pressure are associated with left ventricular hypertrophy and diastolic dysfunction in hypertensive diabetic patients Cardiovascular Diabetology 2006 5 19 10.1186/1475-2840-5-19 16968545 PMC1579206

[B7] Felício JS Pacheco JT Ferreira SR Plavnik F Moisés VA Kohlmann O Ribeiro AB Zanella MT Impaired reduction of nocturnal systolic blood pressure and severity of diabetic nephropathy Exp Clin Cardiol 2007 12 3 157 160 18650998 PMC2323760

[B8] Hansen HP Rossing P Tarnow L Neilsen FS Jensen BR Parving HH Circadian rhythm of arterial pressure and albuminuria in diabetic nephropathy Kidney Int 1996 50 579 85 10.1038/ki.1996.352 8840289

[B9] Lurbe E Redon J Kesani A Pascual JM Tacons J Alvares v Batlle D Increase in Nocturnal Blood Pressure and Progression to microalbuminuria in Type 1 Diabetes New England Journal of Medicine 2002 347 11 797 805 10.1056/NEJMoa013410 12226150

[B10] Lurbe E Redon J Pascual JM Tacons J Alvarez V Batlle D Altered blood pressure during sleep in normotensive subjects with type I diabetes Hypertension 1993 21 227 35 8428785 10.1161/01.hyp.21.2.227

[B11] Moore WV Donaldson DL Chonko AM Ideus P Wiegmann TB Ambulatory blood pressure in type I diabetes mellitus: comparison to presence of incipient nephropathy in adolescents and young adults Diabetes 1992 41 1035 41 10.2337/diabetes.41.9.1035 1499855

[B12] Lafferty AR Werther GA Clarke CF Ambulatory blood pressure, microalbuminuria, and autonomic neuropathy in adolescents with type 1 diabetes Diabetes Care 2000 23 533 8 10.2337/diacare.23.4.533 10857948

[B13] Perloff D Sokolow M Ambulatory blood pressure measurements prognostic implications Arch Malad Coeur Vaissaux 1991 84 221 27 1953280

[B14] Pecis M Azevedo MJ Moraes RS Ferlin EL Gross JL Autonomic dysfunction and urinary albumin excretion rate are associated with an abnormal blood pressure pattern in normotensive normoalbuminuric type 1 diabetic patients Diabetes Care 2000 23 989 93 10.2337/diacare.23.7.989 10895852

[B15] Poulsen PL Hansen KW Mogensen CE Ambulatory blood pressure in the transition from normo to microalbuminuria: a longitudinal study in IDDM patients Diabetes 1994 43 1248 53 10.2337/diabetes.43.10.1248 7926296

[B16] Morgensen CE Chacati A Christensen CK Close CF Deckert T Hommel E Microalbuminuria: An early marker of renal involvement in Diabetes Uremia Invest 1986 9 85 95 10.3109/08860228509088195 3915933

[B17] Felt-Rasmussen B Borch-Johnsen K Deckert T Jensen G Jenden JS Microalbuminuria: An important diagnostic tool Jornal of Diabetes and Its Complications 1994 8 137 145 10.1016/1056-8727(94)90030-2 8086648

[B18] Morgensen CE Christian CK Predicting diabetic nephropathy in insulin-dependent patients N Engl J Med 1984 31 189 193 10.1056/NEJM198407123110204 6738599

[B19] Devereaux RB Reicheck N Echocardiographic determination of left ventricular mass in man. Autonomic valkidation of method Circulation 1997 55 613 618 10.1161/01.cir.55.4.613 138494

[B20] Pickering TG Harshfield GA Kleinert HD Blank S Laragh JH Blood pressure during normal daily activities, sleep, and exercise JAMA 1982 247 9892 9996 10.1001/jama.247.7.992 7057592

[B21] Yasuda G Ando D Hirawa N Umemura S Tochikubo O Effects of Losartan and Anlodipine on Urinary Albumin Excretion and Ambulatory Blood Pressure in Hypertensive Type 2 Dibetic Patients With Overt Nephropathy Diabetes Care 2005 08 1862 68 10.2337/diacare.28.8.1862 16043724

[B22] Lurbe E Redon J Kesani A Pascual JM Tacons J Alvares V The spectrum of circadiam blood pressure changes in type 1 diabetic patients J Hypertension 2001 19 1421 1428 10.1097/00004872-200108000-00010 11518850

[B23] Nakano S Uchida K Kigoshi T Circadian rhythm of blood pressure in normotensive NIDDM subjects: its relationship to microvascular complications Diabetes Care 1991 14 707 805 10.2337/diacare.14.8.707 1954805

[B24] Palmas W Pickering T Teresi J Nocturnal blood pressure elevation predicts progression of albuminuria in elderly people with type 2 diabetes J Clin Hypertens 2008 10 12 20 (Greenwich) 10.1111/j.1524-6175.2007.07170.x PMC8109925 18174766

[B25] Gambardella S Frontoni S Spallone V Increased left ventricular mass in normotensive diabetic patients with autonomic neuropathy Am J Hypertens 1993 6 97 102 8471236 10.1093/ajh/6.2.97

[B26] Tasali E Mokhlesi B Van Cauter E Obstructive sleep apnea and type 2 Diabetes: interacting epidemics Chest 2008 133 496 506 10.1378/chest.07-0828 18252916

[B27] Leitão CB Canani LH Silveiro SP Gross JL Ambulatory Blood Pressure Monitoring and Type 2 Diabetes Mellitus Arquivos Brasileiros de Cardiologia 2007 89 5 347 353 10.1590/S0066-782X2007001700012 18066457

[B28] Felício JS Pacheco JT Ferreira SR Plavnik F Kohlmann O Ribeiro AB Zanella MT Reproducibility of Ambulatory Blood Pressure Monitoring in Hypertensive Patients with Type 2 Diabetes Mellitus Arquivos Brasileiros de Cardiologia 2007 88 2 207 211 10.1590/S0066-782X2007000200012 17384839

[B29] Staessen JA Fagard RH Lijnen PJ Thijs L Hoof RV Amery AK Mean and range of the abulatory pressure in normotensive subjects from meta-analyzes of 23 studies Am J Cardiol 1991 1 723 727 10.1016/0002-9149(91)90529-T 1826069

[B30] Parati G Bilo G Should 24-h ambulatory blood pressure monitoring be done in every patient with diabetes? Diabetes Care 2009 32 298 304 10.2337/dc09-S326 PMC2811450 19875569

[B31] Belletti DA Zacher C Wogen J Effect of cardiometabolic risk factors on hypertension management: a coss-sectional study among 28 physician in the United States Cardiovascular Diabetology 2010 9 7 10.1186/1475-2840-9-7 20122170 PMC2824690

